# Low Formalin Concentrations Induce Fine-Tuned Responses That Are Sex and Age-Dependent: A Developmental Study

**DOI:** 10.1371/journal.pone.0053384

**Published:** 2013-01-07

**Authors:** Ihssane Zouikr, Melissa A. Tadros, Vicki L. Clifton, Kenneth W. Beagley, Deborah M. Hodgson

**Affiliations:** 1 Laboratory of Neuroimmunology, School of Psychology, University of Newcastle, Newcastle, New South Wales, Australia; 2 School of Biomedical Sciences and Pharmacy, University of Newcastle, Newcastle, New South Wales, Australia; 3 Robinson Institute, University of Adelaide, Adelaide, South Australia, Australia; 4 Institute of Health Biomedical Innovation, Queensland University of Technology, Brisbane, Queensland, Australia; Indian Institute of Toxicology Reserach, India

## Abstract

The formalin test is increasingly applied as a model of inflammatory pain using high formalin concentrations (5–15%). However, little is known about the effects of low formalin concentrations on related behavioural responses. To examine this, rat pups were subjected to various concentrations of formalin at four developmental stages: 7, 13, 22, and 82 days of age. At postnatal day (PND) 7, sex differences in flinching but not licking responses were observed with 0.5% formalin evoking higher flinching in males than in females. A dose response was evident in that 0.5% formalin also produced higher licking responses compared to 0.3% or 0.4% formalin. At PND 13, a concentration of 0.8% formalin evoked a biphasic response. At PND 22, a concentration of 1.1% evoked higher flinching and licking responses during the late phase (10–30 min) in both males and females. During the early phase (0–5 min), 1.1% evoked higher licking responses compared to 0.9% or 1% formalin. 1.1% formalin produced a biphasic response that was not evident with 0.9 or 1%. At PND 82, rats displayed a biphasic pattern in response to three formalin concentrations (1.25%, 1.75% and 2.25%) with the presence of an interphase for both 1.75% and 2.25% but not for 1.25%. These data suggest that low formalin concentrations induce fine-tuned responses that are not apparent with the high formalin concentration commonly used in the formalin test. These data also show that the developing nociceptive system is very sensitive to subtle changes in formalin concentrations.

## Introduction

Animal models of pain are crucial for understanding the mechanisms that underlie the maturation of the nociceptive system. These models commonly use behavioural tests such as the hot plate and tail flick tests to assess thermal pain, and the von Frey test to assess mechanical pain. In comparison, the formalin test is a model of acute and persistent pain and involves an inflammatory response with release of neurogenic molecules such as substance P, glutamate and TNFα in the spinal cord [1], [2]. Therefore, this test is a suitable model to investigate the role of the immune system in pain.

Initially described by Dubuisson and Dennis [3], this test is one of the most widely used models in inflammatory pain research [4], [5], [6], [7].The formalin test consists of injecting a small amount of dilute formalin into the plantar (or dorsal) surface of the hindpaw and subsequently assessing the behavioural responses, which can be classified as either flinching or licking [8]. The formalin test elicits a characteristic biphasic response with an early phase starting immediately after the injection and lasting 5 to 10 min followed by a short quiescent interphase and a late phase persisting 60 to 90 min. The early phase is traditionally considered to be the result of direct activation of C fibers by the formalin whereas the late phase is due to the release of inflammatory molecules and increased discharge of dorsal horn neurons [9], [10]. This characteristic biphasic response is also seen in Aδ and C fibers as they both exhibited increased firing activity in response to formalin injection during the early and late phases but not interphase [11], [12].

The response to formalin injection differs according to age. However, PND 3 pups are 10 times more sensitive (exhibited more flinching and licking) to the formalin injection than PND 25 pups [13]. Moreover, 18 -month- old rats exhibited significantly greater flinching and licking in response to formalin injection compared to 24 month- old rats [6]. These data imply that the sensitivity to formalin changes with age as younger animals appear more sensitive to formalin than their adult counterparts.

The available studies that have assessed age differences in formalin responses used high formalin concentrations such as 10% and noted that the licking pattern was absent during the first week [14], [15], [16]. In addition, the characteristic biphasic response was not observed in pups younger than 15 days [13], [15], [16]. However, much remains to be learned about the effects of low formalin concentrations (e.g. <2.5%) on behavioural responses throughout development. Using lower formalin concentrations can help detect subtle differences that are not apparent with higher doses. Therefore the aim of the present experiment was to determine the minimum formalin concentration that enables adequate behavioural responses during four stages of development of the rat: PND 7, 13, 22 and 82.This aim was achieved by performing a dose-response curve using three formalin concentrations at each stage.

## Materials and Methods

### 1. Subjects and Ethics Statement

Wistar rats were obtained from the University of Newcastle vivarium and allowed a two-week period, prior to mating, for acclimatisation to the School of Behavioural Sciences vivarium (Newcastle, Australia). Two adult female breeders were harem housed with one adult male. The male was removed after two weeks and dams were housed individually in custom designed polycarbonate-perspex home boxes (43.5 cm×28.0 cm×12.5 cm cages; Mascot Wire Works, Sydney, Australia). Cages were checked daily until the birth of litters. Twenty Wistar rats (10 males and 10 females) were used in the current study. Following birth (PND 1), pups were left undisturbed until testing days: PND 7, 13, and 22. At PND 22, pups were weaned and segregated into same-sex paired housing (43.5 cm×28.0 cm×12.5 cm) cages and left undisturbed until behavioural testing at PND 82. Pups were randomly allocated across each treatment group such that at the three formalin concentrations; 0.3% (n = 7), 0.4% (n = 7), and at 0.5% (n = 6). All pups were tested at each of the four developmental ages. Rats were maintained in a temperature (21+/−1°C) and humidity (60%) controlled environment, under a 12 h/12 h light-dark cycle (light on 6∶00 h) with food and water available *ad libitum*. All experiments were carried out in accordance with the 2004 National Health and Medical Research Council Australian Code of Practice for the care and use of animals for scientific practice. All procedures were reviewed and approved by the Ethics committee of the University of Newcastle.

### 2. Preparation of Formalin Solution

Formaldehyde (36.5%–38%; Biolab Ltd, Victoria, Australia) and preservative-free saline (Sodium Chloride Injection BP 0.9%, Pfizer, Australia) were used to prepare the stock formalin solutions. 1% formalin was made with 0.1 ml formaldehyde in 9.9 ml saline. The volume of solution injected into the hindpaw was 10 µl for PND 7, PND 13, and PND 22 pups and 50 µl for PND 82 rats. Solutions were mixed one day prior to injection and maintained at room temperature.

### 3. Formalin Testing

Pups were randomly assigned to three groups: at PND 7, pups were subjected to either 0.3% (n = 7), 0.4% (n = 7), or 0.5% formalin (n = 6) injected into the left hindpaw. At PND 13, pups were subjected to either 0.6% (n = 7), 0.7% (n = 7), or 0.8% formalin (n = 6) injected into the right hindpaw. At PND 22, pups were tested either with 0.9% (n = 7), 1% (n = 7), or 1.1% formalin (n = 6) injected into the left hindpaw. At PND 82, rats were tested either with 1.25% (n = 7), 1.75% (n = 7), or 2.25% formalin (n = 6) injected into the right hindpaw. The same pups were tested at each time-point, however the paw that was injected was alternated to allow full recovery of the paw between injections. The different formalin concentrations at different postnatal ages were selected because the sensitivity to formalin-induced pain varies according to age [6], [13], [16], [17], [18]. The choice of formalin concentration range was based on previous studies [4], [16], [17], [19] and in particular the work carried out by Teng and Abbott [13] and adjusted to allow determination of the dose required to produce the required biphasic response. Note that the volume of solution injected into the hindpaw during the first three postnatal weeks was the same (i.e. 10 µl). In adult rats (PND 82), a higher formalin concentration (i.e. 50 µl) was utilised to produce the required behavioural responses [13]. Saline-injected control groups were not required in this study since it has previously been demonstrated that rats which received a subcutaneous injection of 10 µl saline into the plantar surface of the hindpaw do not shake or lick their injected paw when tested at 3, 6, 10, 15, and 20 days of age [16]. Moreover, rats subjected to an injection of 50 µl of saline into the paw displayed no flinching or licking responses [20] nor do they demonstrate c-Fos staining (a marker of neuronal activity) in the superficial dorsal horn [21].

### 4. Testing Apparatus and Behavioural Testing

PND 7 and PND 13 pups were tested in transparent Plexiglas boxes (12 cm (w)×15 cm (l) ×15 cm (h)). A mirror was mounted 45° beneath the floor to allow an unobstructed view of the paws and a camera was mounted to record behavioural responses from the reflection of the mirror. Behavioural responses were recorded on a DVD recorder for one-hour post injection. The testing chamber was maintained at 29–31°C, in order to assist homeostasis in infant rats that have inadequate thermoregulation [18]. PND 22 pups and PND 82 rats were tested in 30 cm×30 cm×30 cm transparent Plexiglas boxes with the same mirror and camera set up as used for PND 7 and PND 13. PND 22 and PND 82 rats were tested at room temperature (22°C). Developmental studies have shown that rat pups do not develop the ability to recognize and interact with the environment until the third postnatal week [22]. Consequently, PND 7, 13 and 22 pups were not acclimated to the testing boxes, whereas PND 82 rats were. PND 82 rats were habituated to the testing conditions by placing them in the testing boxes for 15 min on five consecutive days prior to the testing day, and a 10 min baseline (prior to formalin injection) was also recorded.

On the test day, pups were removed from their housing and weighed. All testing was performed between 9 and 11 am. For testing, pups were randomly selected from each litter for each of the treatment group. The pups were gently restrained and injected subcutaneously into the plantar surface of the pups’ hindpaw using a 31G needle at PND 7, PND13, and PND 22 and using a 30G needle at PND 82. Alternate paws were used for injection at each developmental period to minimize tissue damage. After the formalin injection, the pups were immediately placed in the testing box and monitored for one hour.

### 5. Behavioural Analysis

Flinching and licking, the two main formalin related behavioural responses, were scored according to the technique of Wheeler-Aceto and Cowan [8]. Flinching is described as paw lifting when the response is less intense and as paw shaking when the response is stronger. To score the pain responses, the one hour recording session was divided into 5 min intervals during which the frequency of flinches as well as the duration (in seconds) spent licking the injected paw was scored.

Plots of the mean levels of flinching and licking were generated for each concentration in each age group. Initially, groups were divided by sex and when no sex differences were observed, males and females were combined.

### 6. Statistical Analysis

Data analysis was carried out using the Statistical Package for the Social Sciences for Windows, version 19 (SPSS). Flinching and licking were both analysed as outcome variables. Classically, the formalin response is divided into an early phase and a late phase. In order to assess if any of our group display a biphasic response, we applied different statistics during the early phase (first 5 min) and the late phase (10 to 35 min). The distribution of flinching and licking was positively skewed and variance was not homogenous over time. The effect of the three formalin concentrations on the behavioural responses during the early phase (5 min) was examined using the non-parametric independent test, Jonckheere-Terpstra for ordered alternatives, to deal with the skewness in the data. For the comparison between 5 min and 10 min post-injection, the only differences of interest was whether there was a reduction in flinching response within each formalin concentration, so a non-parametric paired samples test (Wilcoxon) was applied to compare the two time points for each concentration of formalin.

For the second phase (10 min to 35 min), two approaches were adopted. The first calculates the Area Under the Curve (AUC) over time by summing flinching responses from 10 min to 35 min (during the first week, the nature of the response was not biphasic so the AUC was estimated between 10 and 60 min).This analysis determines the temporal pattern of the response. Statistical analysis of AUC was performed using univariate between subjects ANOVA. The second approach determines differences between groups at each time points. In this method, the time response profiles for each formalin concentration were examined using a Linear Mixed Model (LMM) approach since it can handle a failure of the constant variance assumption. A range of residual covariance structures were tested using the Akaike’s Information Criterion (AIC) and Schwarz’s Bayesian Criterion (BIC) information criteria, the autoregressive heterogeneous variance (ARH1) was chosen as it was the best fit. Time was treated as a categorical variable with repeated measures and sex and formalin concentration were between subject variables, all being treated as fixed.

Following a significant interaction effect between time and formalin concentration, follow-up testing at each time period was carried out to identify any differences between formalin concentrations. Paired comparisons were performed using Least Significance Differences (LSD) between the three formalin concentrations at each developmental stage. α was set to 0.05.

## Results

Dose response curves were used to assess the effects of varying concentrations of formalin at different stages of development. We assessed the effects of 3 different concentrations at 4 age points (PND 7, 13, 22, and 82). Each subject was tested at each of the different time points with each of the different concentrations.

### 1. Formalin Flinching Responses at PND 7

**Figure 1:**
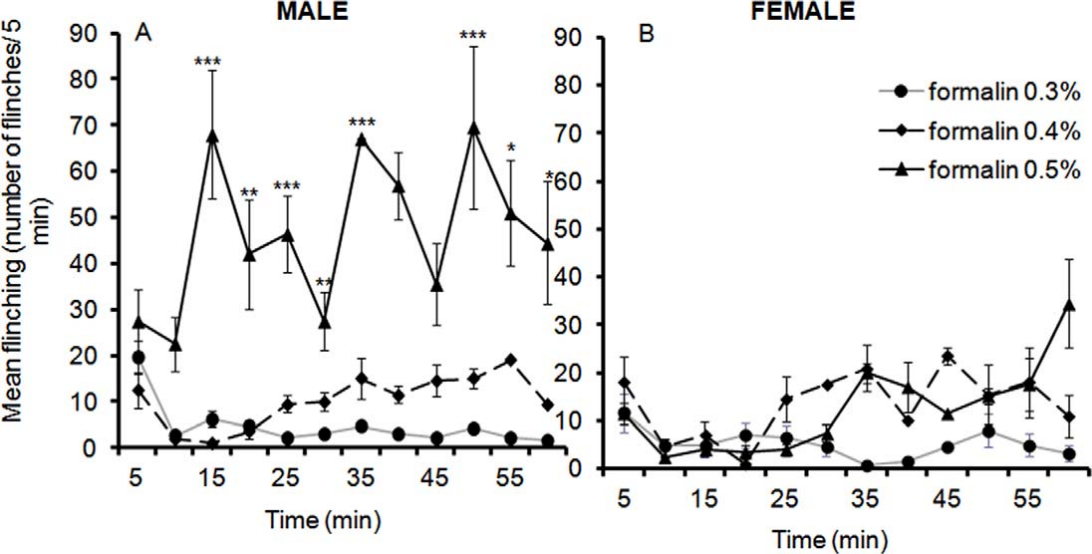
Time course of flinching responses in PND 7 male (A) and female (B) rats in responses to an injection of 0.3%, 0.4%, and 0.5% formalin into the plantar surface of the left hindpaw. Data are presented as mean +/− SE. * *p*<.05; **p<.01; *** *p*<.001, against other groups at the same time point.

Flinching of the affected hindpaw was observed in all animals after formalin injection. LMM analysis of flinching responses from 5 to 60 min indicated a significant three way interaction between sex, formalin dosage and time [*F*(22,75.75) = 3.21, *p*<.001]. In males, the time profiles differed depending on formalin concentration. Pairwise comparisons revealed significant differences between 0.3% and 0.5% and also between 0.4% and 0.5%. In contrast, no significant differences were observed between 0.3% and 0.4% for 10 min through to 40 mins with p value ranging from <.001 through to.004 (Figure 1A).

Analysis of the AUC between 10 min and 60 min revealed a significant effect of formalin dosage on flinching for male pups [*F*(2,7) = 6.38, *p* = .026]. Pairwise comparisons revealed that PND 7 rat pups subjected to 0.5% formalin display significantly higher flinching than those that received 0.3%, *p* = .012 or 0.4%, *p* = .017. There was no significant difference of flinching responses across formalin dosage at 5 min post formalin injection (Jonckheere-Terpstra test for ordered alternatives, standardised test statistic, J^*^ = <.001, *p* = 0.50, one-tailed). In females, analysis of flinching responses revealed no significant main effect of time or formalin dosage on flinches or interaction between them (Figure 1B).

In conclusion, at PND 7 male rat pups displayed a higher frequency of flinching only after 0.5% formalin injection, whereas female pups did not.

### 2. Formalin Licking Responses at PND 7

**Figure 2:**
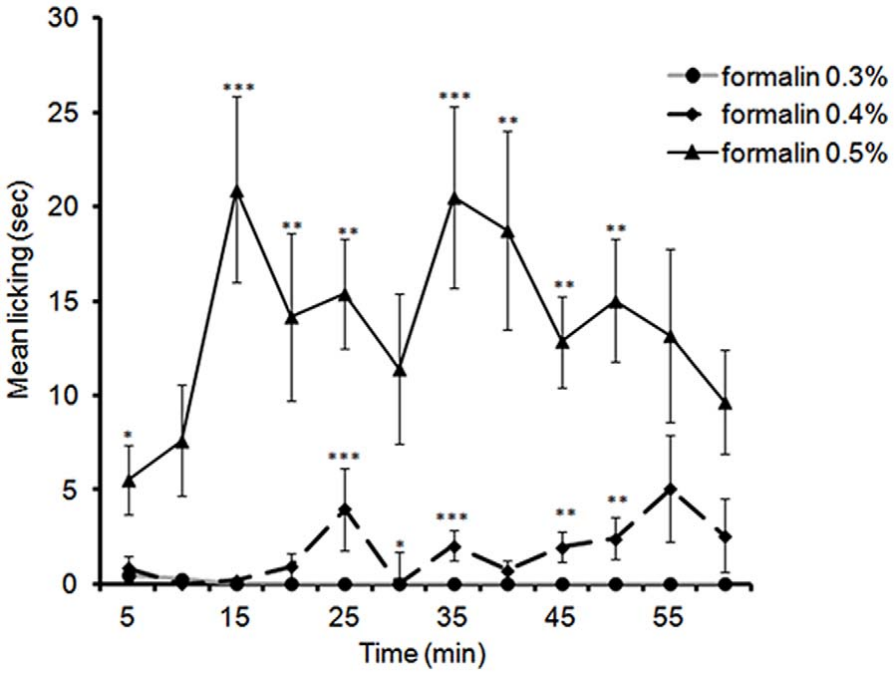
Time course of licking responses in PND7 rats (males and females combined) in response to an injection of 0.3%, 0.4%, and 0.5% formalin into plantar surface of the left hindpaw. Data are presented as mean +/− SE. * *p*<.05; **p<.01; *** *p*<.001, against other groups at the same time point.

Both male and female PND7 rats spent time licking the affected hindpaw after formalin injection. However, there were no sex differences observed, and therefore males and females were combined. LMM analysis of licking responses from 5 to 50 min revealed a significant two way interaction between time and formalin dosage [*F*(18,51.64) = 1.94, *p* = .032]. Pairwise comparisons revealed significant differences between 0.3% and 0.5% and also between 0.4% and 0.5% during all time points except at 30 min where the only significant difference was between 0.3% and 0.5%, *p* = .018 (Figure 2).

Analysis of the AUC between 5 min and 50 min revealed a significant effect of formalin dosage on licking [*F*(2,17) = 25.84, *p*<.001]. Pairwise comparisons revealed that PND7 rat pups subjected to 0.5% formalin display significantly higher licking responses than the ones that received 0.4%, *p*<.001 or 0.3%, *p*<.001(Figure 2). These analyses demonstrate that both male and female rat pups are capable of generating licking behaviours in response to an injection of 0.5%, but not 0.3% or 0.4% formalin. In addition, the profile of these licking behaviours (as observed in the plot of Figure 2) is comparable to the characteristic biphasic response classically observed in older animals with higher formalin concentrations.

### 3. Formalin Flinching Responses at PND 13

**Figure 3:**
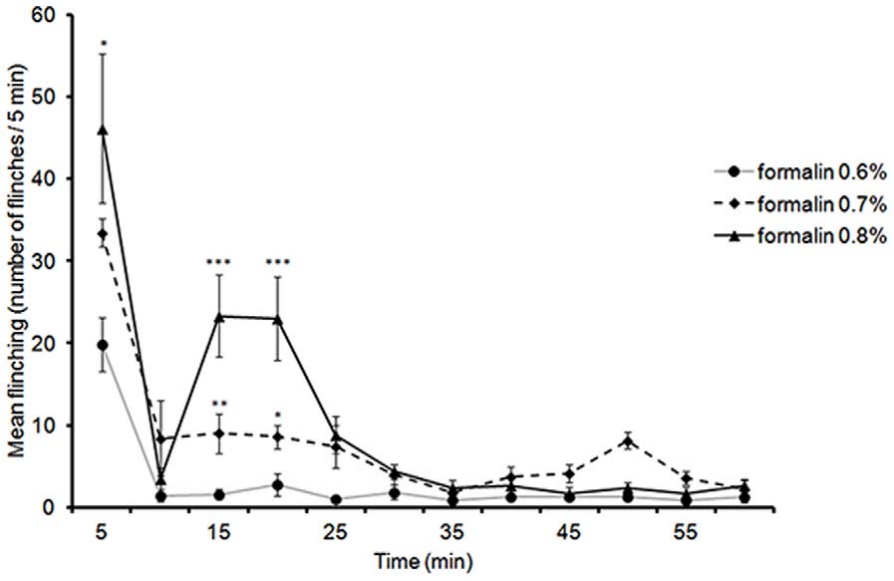
Time course of flinching responses in PND13 rats (males and females combined) in response to an injection of 0.6%, 0.7%, and 0.8% formalin into the plantar surface of the right hindpaw. Data are presented as mean +/− SE. * *p*<.05; **p<.01, *** *p*<.001, against other groups at the same time point.

At this time-point, no sex differences were observed, therefore males and females were combined. All three concentrations examined (0.6%, 0.7% and 0.8%) were able to generate flinching behaviours in PND 13 rats, with the amplitude of the response increasing as the formalin concentration increased. Interestingly, 0.8% formalin was capable of inducing the characteristic biphasic response usually observed in adults during the formalin test (Figure 3). LMM analysis of flinching responses from 5 to 60 min during the second postnatal week revealed a significant two way interaction between formalin dosage and time [*F*(10,46.03) = 3.06, *p* = .005] implying that flinching responses differed over time depending on formalin dosage.

During the early phase (5 min), a significant difference was found between formalin concentrations (Jonckheere-Terpstra test, J^*^ = 2.74, *p* = .003, one-tailed). Pairwise comparisons revealed that 0.7% and 0.8% formalin produced significantly higher flinching than 0.6%, *p* = .0135 and *p* = .047 respectively, with no significant difference between 0.7% and 0.8%, *p* = .189 (Figure 3). To see if the decrease in flinching at 10 min post-formalin injection for each concentration was significant, a non-parametric paired-sample test (Wilcoxon signed rank test) was used and revealed that there was a significant decrease in flinches responses in all concentrations, 0.6% formalin (z = 2.371, *N* – Ties = 7, *p* = .009, one-tailed), 0.7% (z = 2.197, *N* – Ties = 7, *p* = .014, one-tailed), and for 0.8% (z = 2.023, *N* – Ties = 5, *p* = .0215, one-tailed).

This analysis indicates the presence of a biphasic-like profile of flinching at PND13.

Analysis of flinching responses during the late phase (10–35 min) using LMM revealed a significant two-way interaction between time and formalin dosage [*F*(10, 46.03) = 3.06, *p* = .005] suggesting the profile of the flinching response varied according to the formalin concentration injected. Pairwise comparisons revealed that the only significant differences in flinching during the late phase were at 15 min with 0.8% formalin being higher than both 0.6% and 0.7%, *p*<.001 and *p* = .009 respectively, and also higher at 20 min compared to 0.6% and 0.7%, *p* = .001 and.013 respectively. At both time points there were no significant differences between 0.6% and 0.7% (Figure 3).

Analysis of the AUC between 10 and 35 min indicated that formalin dosage had a significant impact on flinching [*F*(2,17) = 6.39, *p* = .009]. Pairwise comparisons indicated that the AUC for 0.8% formalin was significantly higher than the AUC for 0.6%, *p* = .02.

### 4. Formalin Licking Responses at PND 13

At higher dose, all animals displayed licking behaviour during the early phase but this was not statistically significant at any time-point for both males and females (data not shown).

### 5. Formalin Flinching Responses at PND 22

LMM analysis of flinching responses from 5 to 60 min revealed a significant three way interaction between sex, formalin dosage, and time [*F*(22, 45.64) = 4.10, *p*<.001], indicating that the varying formalin concentrations impacted upon male and female rats differently.

**Figure 4:**
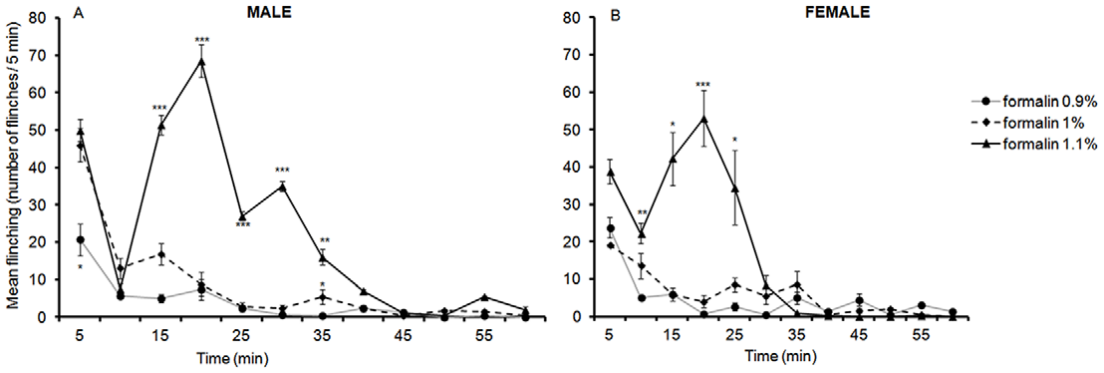
Time course of flinching responses in PND22 male (A) and female (B) rats in response to an injection of 0.9%, 1%, and 1.1% formalin into the plantar surface of the left hindpaw. Data are presented as mean +/− SE. * *p*<.05; **p<.01; *** *p*<.001, against other groups at the same time point.

In males, pairwise comparisons revealed significant differences between 0.9% and 1% as well as 1% and 1.1% for times 15 min through to 30 min and at 55 min with p value ranging from <.001 through to.003. A significant difference was also detected between 0.9% and 1% at both 5 min and 35 min post formalin injection (*p* = .016 and *p* = .026 respectively) and between 1% and 1.1% at 40 min (*p* = .047) (Figure 4A).

Analysis of the AUC between 10 min and 60 min revealed a significant effect of formalin dosage on flinching [*F*(2,7) = 101.01, *p*<.001]. Pairwise comparisons revealed that male PND 22 rats subjected to 1.1% formalin displayed significantly higher flinching compared to those who received 1% (*p*<.001) or 0.9% formalin (*p*<.001). These analyses indicated that although 0.9% and 1% formalin injected into the hindpaw were capable of generating a response, the maximal response was observed with 1.1% formalin. In addition, 1.1% formalin was able to produce the characteristic biphasic response of flinching responses in male PND 22 rats.

In females, pairwise comparisons revealed a significant difference in the frequency of flinching between 0.9% formalin and 1.1% at 10, 15, 20, and 25 min with p values ranging from.007 through to.029. Pairwise comparisons also revealed a significant difference in flinching responses between 1% and 1.1% formalin at 10 min (*p* = .029) and at 15 min (*p* = .001) (Figure 4B).

Analysis of the AUC between 10 min and 60 min revealed a significant effect of formalin dosage on flinching [*F*(2,7) = 6.75, *p* = .023]. Pairwise comparisons revealed that female rats which received 1.1% formalin exhibited significantly higher flinching responses than the one that received 0.9% (*p* = .01). Similarly to males, 1.1% formalin produced the maximal response in female rats, and again had a profile comparable to that of the characteristic biphasic response.

### 6. Formalin Licking Responses at PND 22

Licking responses were observed in both male and female PND 22 rats throughout the one hour recording period. LMM analysis of licking responses between 5 and 60 min revealed a significant three way interaction between sex, formalin dosage and time [*F*(22,26.91) = 2.09, *p* = .034], suggesting that again the varying concentration of formalin had differing responses on both sexes.

**Figure 5:**
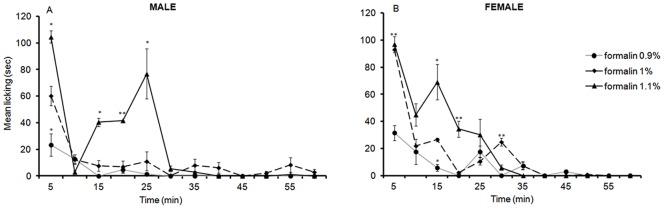
Time course of licking responses in PND22 male (A) and female (B) rats in response to an injection of 0.9%, 1%, and 1.1% formalin into the plantar surface of the left hindpaw. Data are presented as mean +/− SE. * *p*<.05; **p<.01, against other groups at the same time point.

In males, pairwise comparisons revealed a significant difference in the time spent licking between 0.9% and 1.1% formalin with animals subjected to 1.1% formalin injection displaying significantly longer licking times at 5, 15, 20, and 25 min with p values ranging from.008 to.02. In addition, pairwise comparisons revealed a significant difference in licking responses between 1% and 1.1% with rats subjected to 1.1% formalin injection displaying significantly higher licking time at 15, 20, and 25 min with p value ranging from.007 to.017 (Figure 5A).

Analysis of the AUC between 10 min and 30 min revealed a significant effect of formalin dosage on licking in male rats [*F*(2,7) = 6.1, *p* = .029]. Pairwise comparisons revealed that males who received 1.1% formalin exhibited significantly higher licking responses than those who received 0.9%, *p* = .014 or 1%, *p* = .018. In addition, the profile of the plot of time spent licking was again comparable to the characteristic biphasic response.

In females, analysis of licking responses during the first 35 min revealed a significant difference in licking duration between 0.9% and 1.1% formalin. Females subjected to 1.1% formalin injection spent significantly more time licking their hindpaw at 5, 15, and 20 min (*p = *.001; *p* = .018 and *p* = .004 respectively). Likewise, female rats that received 1% formalin also displayed significantly more time licking compared to their counterparts that received 0.9% at 5 (*p* = .005) and 30 min (*p* = .002). In addition, 1.1% formalin injection produces significantly more licking compared to 1% at 20 and 30 min (*p* = .017 and *p* = .004 respectively) (Figure 5B).

Analysis of the AUC between 10 min and 35 min revealed a significant effect of formalin dosage on licking [*F*(2,7) = 7.3, *p* = .019]. Pairwise comparisons revealed that the only significant difference was between 0.9% and 1.1% formalin with 1.1% formalin producing significantly higher licking responses than 0.9% during the second phase (*p* = .007).

In conclusion, both male and female rats displayed licking responses to all three concentrations of formalin injected. However, 1.1% formalin produced the maximum response that also had a profile similar to the characteristic biphasic response.

### 7. Formalin Flinching Responses at PND 82

At this time-point, no sex differences were observed, therefore males and females were combined. All three concentrations of formalin (1.25%, 1.75% and 2.25%) were capable of generating flinching responses in both male and female rats after injection into the hindpaw. LMM analysis of flinching responses between 0 and 60 min revealed a significant two way interaction between formalin dosage and time [*F*(22,66.64) = 1.99, *p = *.016].

During the early phase (5 min), no significant differences were found between formalin concentrations (Jonckheere-Terpstra test, J^*^ = .208, *p* = .417, one-tailed). The decrease in flinching responses 5 min after formalin-injection was significant for all formalin concentrations (Wilcoxon paired-sample test), 1.25% formalin (z = 2.36, *N* – Ties = 7, *p* = .009, one-tailed), 1.75% (z = 2.36, *N* – Ties = 7, *p* = .009, one-tailed), and for 2.25% (z = 2.20, *N* – Ties = 6, *p* = .014, one-tailed).

**Figure 6:**
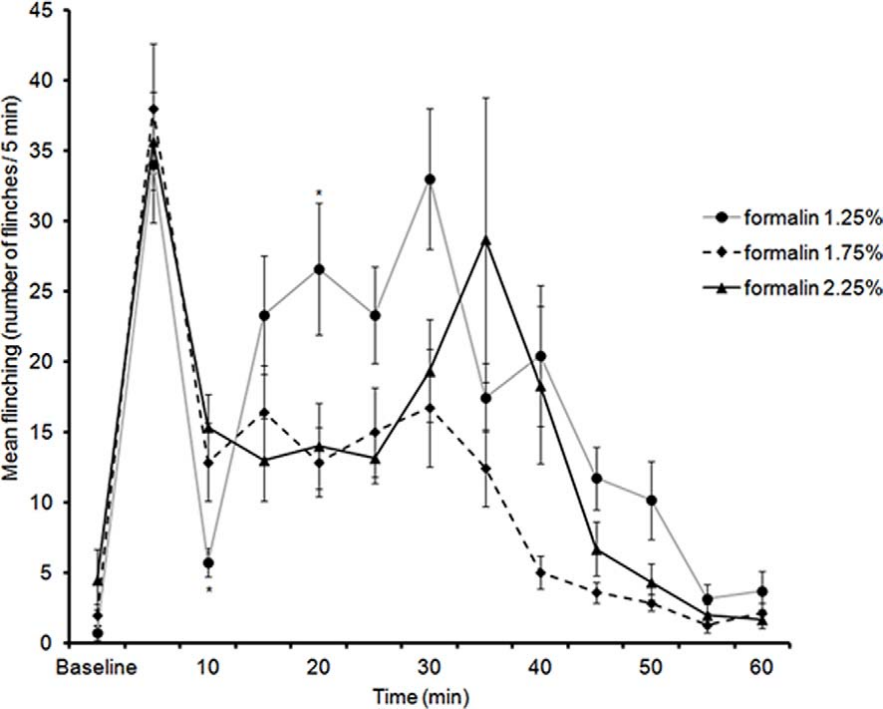
Time course of flinching responses in PND82 rats (males and females combined) in response to an injection of 1.25%, 1.75%, and 2.25% formalin into the plantar surface of the right hindpaw. Data are presented as mean +/− SE. * *p*<.05, against other groups at the same time point.

Analysis of flinching responses during the late phase (10–35 min) using LMM revealed a significant two-way interaction between time and formalin dosage [*F* (10, 33.88) = 2.84, *p* = .011]. Pairwise comparisons revealed that the only significant differences in flinches during the second phase were at 10 min between 1.25% and 2.25% (*p* = .048) and at 20 min between 1.25% and 1.75% (*p* = .043) (Figure 6). In summary, although all three concentrations produced a biphasic response, only 2.25% formalin induced an interphase similar to that observed in previous studies [7].

### 8. Formalin Licking Responses at PND 82

Both male and female PND 82 rats spent time licking hindpaw after injection of formalin, regardless of the concentration. However, LMM analysis of licking responses indicated a significant three way interaction between sex, formalin dosage, and time [*F*(22,30.76) = 2.38, *p* = .013], suggesting that the concentration had different effects on the amplitude of the response for males and females.

**Figure 7:**
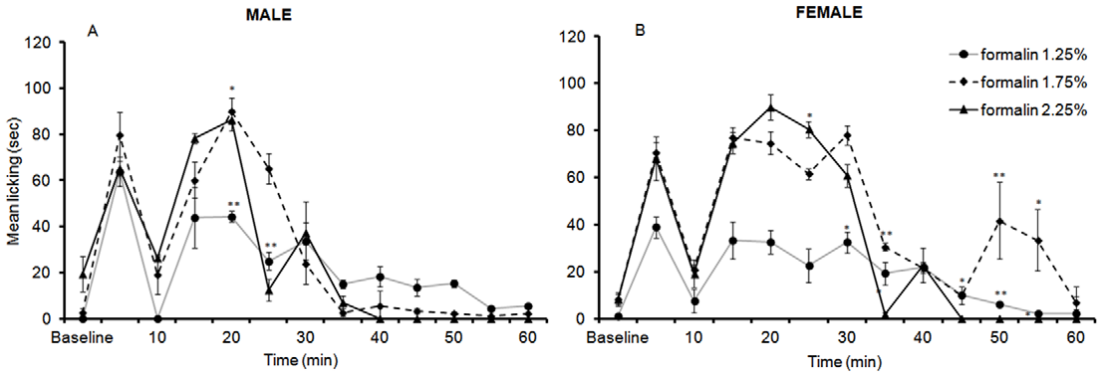
Time course of licking responses in PND82 male (A) and female (B) rats in response to an injection of 1.25%, 1.75%, and 2.25% formalin into the plantar surface of the right hindpaw. Baseline is the time prior to formalin injection (10 min). Data are presented as mean +/− SE. * *p*<.05, **p<.01, against other groups at the same time point.

In males, analysis of licking responses during the first 35 min indicated that both 1.75% and 2.25% formalin produces significantly greater licking responses than 1.25% at 20 min (*p* = .003, and *p* = .016 respectively). At 25 min the time spent licking in response to 2.25% formalin was significantly longer than that induced by 1.75% formalin, *p* = .005 (Figure 7A).

In females (Figure 7B), during the early phase (5 min), no significant differences were observed between formalin concentrations (Jonckheere-Terpstra test, J^*^ = 1.36, *p* = .08, one-tailed). The decrease in flinching responses 5 min post formalin-injection was only significant for 2.25% formalin (Wilcoxon paired-sample test; z = 1.82, *N* – Ties = 4, *p* = .034, one-tailed).

Analysis of licking responses during the late phase (10–35 min) using LMM revealed a significant two-way interaction between time and formalin dosage [*F* (10, 20.86) = 3.59, *p* = .007]. Pairwise comparisons revealed that 1.75% formalin produced significantly longer time spent licking compared to 1.25% formalin at 30, 35, 50, and 55 min *(p* = .010, *p* = .01, *p* = .004, and *p = *.012, respectively). In addition, 2.25% formalin evoked significantly more licking in rats than 1.75% formalin at 35, 45, 50, and 55 min (*p* = .004, *p* = .01, *p* = .004, *p* = .012, respectively) or 1.25% formalin at 20 min, *p* = .01 (Figure 7B).

In summary, these findings indicate that for males, significant biphasic response was observed at all doses but it was significantly more pronounced at the two higher concentrations. Moreover, for females, the same pattern of results was observed. Although interestingly there was a small late phase peak at 50 min in females not apparent in males.

## Discussion

The present study demonstrates that the behavioural responses (flinching and licking) to formalin vary depending on formalin concentration as well as age and sex. A major finding of the current study is that subcutaneous injection of 0.8% formalin into the plantar surface of the hindpaw elicited a biphasic response in PND 13 rats. Another prominent finding is that at PND 7, an injection of 0.5% formalin evoked a biphasic-like pattern in licking responses. We have also demonstrated that at PND 22, the characteristic biphasic response of both licking and flinching was only observed with 1.1% formalin, indicating that the nociceptive system is less sensitive at this stage of development. In adulthood (PND 82), all three formalin concentrations produced a well-defined biphasic response with an interphase lasting 15 to 20 min for both 1.75% and 2.25% but not 1.25%. In addition, sex differences in flinching were observed during the first and third postnatal week whereas sex differences were observed in licking behaviours during the third postnatal week and in adulthood. This implies that the appearance of sex differences in these pain responses is developmentally regulated.

In summary, our findings suggest that low formalin concentrations produce subtle changes in the formalin behavioural responses throughout development. However, small variations in these concentrations are more likely to be detected by the developing nociceptive system during the first two postnatal weeks. This system becomes less sensitive from the beginning of the third week where higher formalin concentrations are needed to produce the characteristic biphasic response.

### Sex Differences in the Formalin Behavioural Responses

An increasing number of researchers have reported that males and females differ in their sensitivity to pain [23], [24], [25]. The general consensus is that female rats display greater nociceptive behaviour than their male counterparts [26], [27], [28], [29], [30]. These differences can be attributed, in part, to gonadal steroids [31]. For instance, male rats that received an intracerebroventricular injection of estradiol exhibited significantly more licking in response to formalin injection compared to their matched control group [32]. In addition, estrogen may act on the brain regions involved in formalin responses differently during the various stages of the estrous cycle and throughout development. There are two peaks in vaginal opening which occur in Wistar rats: one at PND 34 and one at PND 39 [33], with the first proestrus occurring five to seven days following the vaginal opening [34]. In our current study, we observed a sex difference in flinching responses at PND 7 and PND 22, with males displaying higher and longer flinching than females in response to an injection of 0.5% (PND 7) or 1.1% (PND 22). In comparison, for licking responses, sex differences were observed at PND 22 and PND 82 with males being more susceptible to higher formalin concentration than females. This suggests that both the development and the stage of the estrous cycle may have an effect on the behavioural responses to formalin injection.

### Neonatal Formalin Behavioural Responses (PND7 and 13)

It has been reported that adult female rats flinch more frequently than males in response to formalin injection [26], [29]. These findings differ from ours, in that we observed more flinching in males than females at PND 7 (0.5% formalin injection, Figure 1). This discrepancy may well depend on the different formalin concentrations used (0.3–0.5% vs. 10%) but also on the age of the tested animals (neonates vs. adults). It is noteworthy that 10% formalin injection evoked more flinching and licking in females than in males. In contrast, 0.1% formalin produced higher flinching and licking responses in males [35]. This latter finding demonstrates that the formalin concentration is a critical factor in demonstrating the sexual dimorphism in formalin related responses.

Interestingly, 0.4% and 0.5% formalin produce notable licking responses while 0.3% formalin evokes no licking responses at PND 7 (Figure 2). This finding is in contrast with several reports in the literature suggesting that the licking response is infrequent or absent in pups younger than 10 days of age [14], [15], [16]. These studies used formalin concentration ranging from 1% to 2.25% whereas we used 0.3%–0.5% formalin. McLaughlin and co-workers [36] reported that PND 3 rats were able to lick their paw in response to 15% formalin injection. Thus, it appears varying concentrations of formalin can have widely different effects on the behavioural responses in neonatal rats. One possibility is that the immaturity of sensory processing within the brainstem leads to lower thresholds for excitation and sensitization. In addition, large cutaneous receptive fields in neonates [37] may also lead to this hyperexcitability. However, whether PND 7 pups are able to integrate and analyse the noxious stimulus resulting from 0.5% formalin injection remains to be investigated.

The current study also demonstrates that at PND 13, 0.8% formalin evokes a biphasic response (Figure 3). These findings are in contrast with those of Teng [13] and Guy [16] who reported that the characteristic biphasic response is not evident in animals prior to PND 15. There are many possible reasons for this discrepancy: it may be due to differences in formalin dosage as well as the age of the tested animals. Teng used 0.5%, 1%, and 2% at PND 15 and Guy used 1% at PND 6 and 2.5% in PND 15 rats. Using lower formalin concentrations, we were able to see fine-tuned responses that might be absent with higher dosage. Higher formalin concentrations do not always produce greater nociceptive responses. For instance, a “saturating state” of maximum responses may be reached. Prior studies have demonstrated that an injection of 5% formalin into the plantar rat paw produces higher flinching and licking responses compared to 10% [20]. Additionally, the supraspinal descending inhibitory system develops during the second postnatal week [38]. The functional maturation of this system would, in turn, affect the nociceptive behaviours generated in response to the formalin injection.

Another possibility that must be considered is the difference in rat strains. Both Teng and Guy used Long-Evans rats whereas we used Wistar rats. It has been previously shown that Lewis rats display less pain behaviour than Fisher rats during the late phase of the formalin test [39]. Whether similar differences in terms of sensitivity to noxious stimuli exist between Long-Evans and Wistar rats remain to be ascertained.

### Repetitive Exposure to Formalin Alters the Pattern of the Biphasic Response

The neonatal period is a time of considerable structural and functional plasticity within the neuronal circuitry as well as the developmentally regulated expression of key molecules that modulate nociception [40], [41]. To date, several animal studies have demonstrated that neonatal exposure to noxious stimulation results in altered pain responses later in life [42], [43], [44], [45]. For instance, neonatal rat pups (PND 0) subjected to carrageenan (1%) displayed hyperalgesia to thermal stimuli in adulthood (PND 40) [46]. Moreover, rat pups repeatedly subjected to needle prick stimulation during the first postnatal week exhibited decreased withdrawal latencies to intense heat in adulthood [47]. In our paradigm, all rats underwent multiple formalin injections (i.e. at PND 7, 13, 22, and 82). Although we took appropriate measures to allow full recovery between formalin exposures, it is possible that the repetitive exposure to formalin had an effect on the animals tested in this study. These effects could include hyper-innervation of the injected area as well as disruption of the nociceptive neuronal circuitry and thus produce the biphasic response earlier than expected.

In conclusion, we have demonstrated that rats displayed the characteristic biphasic response earlier than previous studies, which may result from the neonatal challenge with formalin. Further studies focusing on specific periods of development would be required to fully understand and characterise the behavioural responses to formalin throughout development.

### Weanling and Adult Formalin Responses (PND 22 & PND 82)

During the third week of development (PND 22), the nociceptive system was less sensitive and higher formalin concentrations were necessary to see a biphasic response. At this stage, 1.1% evoked a biphasic response in both flinching and licking that was not evident with 0.9% or 1% formalin. In addition, sex differences were observed in flinching and licking. At PND 82, both 1.75% and 2.25% produced an interphase lasting 15 min whereas 1.25% failed to do so. This interphase appears only at PND 82 and was absent at the earlier time-points examined in this study. This is consistent with previous findings where the interphase was seen only at 35 days of age [48].

In conclusion, using lower formalin doses, we were able to see fine-tuned responses not observed in previous studies. This includes the appearance of licking patterns during the first postnatal week and the occurrence of the characteristic biphasic response as early as PND 13. These findings add valuable insights regarding how the nociceptive system responds to different formalin concentrations over the postnatal developmental period. More importantly, this study emphasizes the importance of using appropriate doses of formalin in order to elicit the characteristic biphasic response.
